# The Role of the Mediterranean Diet in Assisted Reproduction: A Literature Review

**DOI:** 10.3390/nu16162807

**Published:** 2024-08-22

**Authors:** Dimitris Baroutis, Theodoros Kalampokas, Eleni Katsianou, Alexandros Psarris, George Daskalakis, Konstantinos Panoulis, Makarios Eleftheriades

**Affiliations:** 1 Aretaieio Hospital, 2nd Department of Obstetrics & Gynecology, National and Kapodistrian University of Athens, 11528 Athens, Greece; 2 Alexandra Hospital, 1st Department of Obstetrics & Gynecology, National and Kapodistrian University of Athens, 11528 Athens, Greece

**Keywords:** Mediterranean Diet, infertility, fertility, assisted reproductive techniques, assisted reproduction, in vitro fertilization

## Abstract

The Mediterranean Diet, characterized by high consumption of plant-based foods, olive oil, moderate intake of fish and poultry, and low consumption of red meat and processed foods, has been suggested to improve assisted reproductive technology (ART) outcomes. This narrative review aimed to summarize and synthesize the evidence from observational studies on the associations between preconception adherence to the Mediterranean Diet and ART outcomes. PubMed/MEDLINE, Embase, ScienceDirect, Google Scholar, and Web of Science databases were searched to identify relevant studies. Seven observational studies (*n* = 2321 women undergoing ART) were included. Adherence to the Mediterranean Diet was assessed using food frequency questionnaires with 6–195 items. Three studies found that higher Mediterranean Diet scores were associated with improved clinical pregnancy rates (OR 1.4, 95% CI 1.0–1.9; RR 1.98, 95% CI 1.05–3.78) or live birth rates (RR 2.64, 95% CI 1.37–5.07). Two studies showed a positive effect on embryo yield (*p* = 0.028) and ovarian response. However, two studies reported no significant associations with ultimate ART success, and four studies found no effects on oocyte and embryo number or quality. The heterogeneity in study designs, Mediterranean Diet assessment methods, and ART protocols limited the strength of conclusions. Evidence for the effects of greater adherence to the Mediterranean Diet on ART outcomes is limited but promising. Future research should focus on conducting randomized controlled trials with standardized Mediterranean Diet assessment methods to establish causal relationships between Mediterranean Diet adherence and ART outcomes, and to elucidate potential mechanisms of action.

## 1. Introduction

The Mediterranean Diet, developed in countries around the Mediterranean, emphasizes a high consumption of plant foods such as fruits, vegetables, whole grains, legumes, nuts, and olive oil, combined with moderate amounts of fish, poultry, eggs, and dairy products [1,2,3]. The Mediterranean Diet’s value in supporting and improving human health is indisputable, as it exhibits a significant protective effect against cardiovascular diseases [1,4], diabetes mellitus, metabolic syndrome [5], and certain types of cancer [1,6]. Recent research highlights the Mediterranean Diet as a useful tool for promoting fertility and improving assisted reproduction results, even supporting the position that the Mediterranean Diet should be recommended to infertile couples who resort to assisted reproduction methods (ART) for having a child [7].

Infertility, defined by the World Health Organization as a disease, plagues up to 15% of reproductive-age couples, with its incidence and prevalence increasing [8,9,10,11]. Assisted reproduction, a branch of reproductive medicine science, includes procedures such as IVF and ICSI and serves infertile couples to conceive and create a complete family [8,9]. Although assisted reproduction techniques (ART) have greatly contributed to infertility treatment, they remain expensive and invasive methods, with possible complications and live and viable birth rates per ART/IVF cycle of only 30% [12]. Therefore, it becomes obvious that there is a need to improve these techniques per se and the adjunctive treatment of infertility in couples seeking treatment through ART with complementary, easily applicable measures such as dietary intervention to further improve both the final and intermediate results of assisted reproduction.

The majority of studies on the Mediterranean Diet have examined and highlighted the benefits of specific components and foods of the Mediterranean Diet in improving several parameters of human fertilization. These include:

- Semen quality: olive oil, rich in monounsaturated fats and antioxidants, and fish, high in omega-3 fatty acids, have been associated with improved sperm parameters [13,14,15,16,17].
- Egg quality and development: fruits and vegetables, abundant in antioxidants, and whole grains, high in fiber and B vitamins, may contribute to better oocyte quality and maturation [13,18,19,20,21,22,23,24,25,26].
- Embryo quality: nuts, containing antioxidants and healthy fats, and legumes, rich in folate and plant-based protein, have been linked to improved embryo morphology and development [27,28,29,30,31].
- Optimization of conditions for implantation: the anti-inflammatory properties of the Mediterranean Diet, derived from its overall composition of plant-based foods and healthy fats, may contribute to a more favorable endometrial environment for implantation [13].

These findings collectively suggest that the Mediterranean Diet’s components may have synergistic effects on various aspects of fertility and assisted reproduction outcomes [13].

The Mediterranean Diet is rich in antioxidants, polyunsaturated fatty acids (such as ω3 & ω6 fatty acids), monounsaturated fatty acids (PUFAs & MUFAs), fiber, and vitamins C, E, and the vitamin B complex [32,33,34,35,36], which contribute to reproductive health [5] through mechanisms that include reducing inflammation [36], increasing insulin sensitivity [13], and protecting against oxidative stress and its damage [23,24,25,26,27,28,37,38].

Despite the Mediterranean Diet being defined as an entity since the 1960s [39], the investigation of its relationship with the improvement of human fertility and especially with assisted reproduction has only recently gained more interest. The fact that the majority of the existing literature investigates the nutritional elements and substances individually should be emphasized, as not to overlook their possible synergistic actions in a wider context by considering them as a whole that constitutes a Diet—Nutritional Pattern/Model. Therefore, the holistic examination of the Mediterranean Diet and the highlighting of the beneficial role of the individual components and foods that make it up through the overall combination and interaction is of particular research interest with possible further clinical applications can bridge an important knowledge gap.

Consequently, this narrative review focuses on summarizing the existing knowledge and literature on one of the most popular diets, arguably the Mediterranean Diet, regarding its role and impact on the outcome of assisted reproduction.

## 2. Materials and Methods

This narrative review was conducted to synthesize and analyze the existing literature on the Mediterranean Diet and its role in assisted reproduction. While we followed a structured approach to literature search and selection, this review was not conducted according to PRISMA guidelines, as it is not a systematic review or meta-analysis.

### 2.1. Search Strategy

A comprehensive literature search was performed using multiple databases and online platforms including the following: PubMed/MEDLINE, Embase, Google Scholar, ScienceDirect, Scopus, and Web of Science (Clarivate). The search was conducted from October 2022 to July 2023. The following search algorithm was used:

(ICSI OR “Intracytoplasmic sperm injection” OR IVF OR fertility OR infertility OR ART OR “assisted conception” OR “assisted reproductive technology” OR “assisted reproductive” OR “assisted reproduction”) AND (“mediterranean diet”)

Additionally, the ‘snowball literature searching method’ was employed to identify additional relevant sources from the reference lists of selected articles.

### 2.2. Study Selection

The selection of included studies was carried out based on their relevance to the subject in terms of their title and abstract, followed by a full-text examination. The inclusion criteria were:

1. Studies examining the Mediterranean Diet as a whole when used by infertile couples seeking ART.
2. Studies dealing with ART for the purpose of IVF/ICSI.
3. Published original research studies.
4. Studies including methods for evaluating adherence to the Mediterranean Diet.

Exclusion criteria were:

1. Studies focusing only on specific ingredients, vitamins, or trace elements.
2. Studies focusing only on cryopreservation, embryo storage, donation, or surrogacy.
3. Studies focusing only on specific pathological conditions associated with infertility (e.g., endometriosis, polycystic ovary syndrome).
4. Literature reviews, systematic reviews, and case reports.

There was no time limit applied, and only English language publications were considered.

### 2.3. Data Extraction and Synthesis

Two reviewers independently extracted data from the selected studies using a standardized form. The extracted information included the following: first author, publication year, country, study design, sample size and characteristics, ART protocol, method of evaluating Mediterranean Diet adherence, duration of study and follow-up period, main results, and confounding factors considered.

Our review included both women undergoing their first IVF cycle and those in subsequent cycles. We evaluated the association between adherence to the Mediterranean Diet before ART therapy and both intermediate outcomes (number and quality of oocytes at retrieval, number and quality of embryos, embryo transfer, implantation) and final results of assisted reproduction.

### 2.4. Quality Assessment

While a formal quality assessment of included studies was not conducted due to the narrative nature of this review, we critically evaluated each study’s methodology, sample size, and potential biases during our analysis and interpretation of results. Our evaluation considered the following aspects:

- Study design: we assessed whether the design (cohort, cross-sectional) was appropriate for addressing the research question.
- Sample size: we considered the number of participants in each study.
- Mediterranean Diet assessment methods: we evaluated the tools used to measure diet adherence, including the number of food items assessed and whether the tools were validated.
- ART protocol: we noted the type of ART protocol used and whether it was clearly described.
- Outcome measures: we assessed the clarity and relevance of the reported outcomes.
- Confounding factors: we examined which potential confounders were considered in each study.
- Reporting of results: we assessed the clarity and completeness of the reported findings.

These evaluations informed our interpretation of results and discussion of study limitations, allowing us to contextualize the findings within the broader literature on Mediterranean Diet and ART outcomes.

## 3. Results

### 3.1. Study Features & Demographics

After a thorough search of the above-mentioned databases, seven studies were found that met the inclusion criteria in the present literature review [40,41,42,43,44,45,46], of which six were cohort studies, while one was a cross-sectional study (Noli et al.) [46], which in total included 2,321 women with infertility under ART therapy [8,9]. Individual sample sizes ranged from 161 to 590 women. The main characteristics of the studies in the present literature review are summarized in Table 1.

### 3.2. Evaluation of Adherence/Following of the Mediterranean Diet

The assessment of adherence to the Mediterranean Diet in the included studies was carried out through food frequency questionnaires (FFQs), which were used to calculate standardized scores. These scores quantify the degree to which an individual’s diet aligns with the key components of the Mediterranean dietary pattern. Higher scores indicate greater adherence to the Mediterranean Diet principles. The scoring systems varied across studies but all aimed to capture the essential elements of the Mediterranean Diet, such as high consumption of fruits, vegetables, whole grains, and olive oil, moderate consumption of fish and poultry, and low consumption of red meat and processed foods. Twigt et al. [41] used a 6-food group questionnaire in the form of “YES or NO” responses to questions of consumption, which was based on the National Dutch Dietary Guidelines [47], but this modified questionnaire had not been previously validated and evaluated. The studies of Karayiannis et al. [42] and Gaskins et al. [43] used the MedDietScore [47] with a scale of 0–55, while the studies of Ricci et al. [44] and Sun et al. [45] used a variant of the MDS with a scale of 0–9 [1]. Vujkovic et al. [40] used a FFQ with analysis based on main dietary characteristics to infer adherence to certain dietary patterns, including the Mediterranean Diet, with a score of 0–8. Finally, Noli et al. [46] used a validated and recognized 78-food-groups-and-items FFQ [48]. The individual FFQs showed great heterogeneity among themselves, which makes their comparison very difficult and challenging. Indicatively, the agreement rate of MedDietScore [47] and of MDS [1] is about 65% [49].

The degree of adherence to the Mediterranean Diet varied across the studies, both in terms of how it was measured and the levels of adherence observed. In the study by Karayiannis et al. [42], using the MedDietScore (range 0–55), the mean score was 32.2 ± 4.5, with 29% of participants in the highest adherence tertile (score ≥ 36). Gaskins et al. [43], also using the MedDietScore, reported a median score of 33 (interquartile range: 30–36). In contrast, Ricci et al. [44], using a 0–9 point scale, found a median score of 4 (interquartile range: 3–5), indicating moderate adherence. Sun et al. [45], using a modified 0–8 point scale, reported a mean score of 4.8 ± 1.3. Noli et al. [46], using a 0–9 point scale, observed a median score of 4 (interquartile range: 3–5). These variations in adherence levels, coupled with the different scoring systems used, further complicate the comparison of results across studies and underscores the need for standardized assessment methods in future research.

### 3.3. ART Protocols

The ART protocols followed varied among the studies in this review, including GnRH agonists and antagonists, as well as other hormonal adjuvant regimens, based on the experience and region of each IVF center.

### 3.4. Results—Associations of the Mediterranean Diet and ART Outcomes

The association and role of the Mediterranean Diet with assisted reproduction for each of the seven studies is summarized below and aggregated in Table 2:

1. The study of Vujkovic et al. (2010) [40] found that higher adherence to a Mediterranean Diet among couples undergoing IVF/ICSI was associated with increased odds of clinical pregnancy (OR 1.4, 95% CI 1.0–1.9). However, adherence to the Mediterranean Diet was not associated with embryo quality.
2. The study of Twigt et al. (2012) [41] reported that each one-unit increase in the mother’s preconception nutritional risk score assessing adherence to the Dutch Dietary Guidelines [49] was associated with a 65% increase in the likelihood of ongoing pregnancy—pregnancy progression (ultrasound detection of heart rate at 10 weeks of gestation) after a 1st ART cycle.
3. The study of Karayiannis et al. (2018) [42] observed that higher Mediterranean Diet scores were associated with increased clinical pregnancy rates (RR 1.98, 95% CI 1.05–3.78) and live birth rates (RR 2.64, 95% CI 1.37–5.07) among women under 35 years of age undergoing their 1st cycle of in vitro fertilization. No associations were observed between the Mediterranean Diet and the number of eggs or the quality of the embryos.
4. The study of Gaskins et al. (2019) [43] found that the Mediterranean Diet was associated with improvement in live birth rates above the first quartile of adherence (0.44, 95% CI: 0.39–0.49, *p* < 0.05), versus the first quartile: (0.31, 95% CI: 0.25–0.39, *p* < 0.05). However, there was no further improvement in live birth rates above the second quartile. No significant correlation was noted with clinical pregnancy rates.
5. The study of Ricci et al. (2019) [44] reported no significant associations between Mediterranean Diet adherence score and oocyte number, embryo quality, clinical pregnancy, or live birth rates among women undergoing IVF. A marginally lower risk of failure to achieve clinical pregnancy for the intermediate Mediterranean Diet scores in women >35 years was noted (aRR 0.84, 95% CI 0.71–1.00, *p* < 0.05) with no associated increase in live birth rates.
6. The study of Sun et al. (2019) [45] found that higher Mediterranean Diet scores were associated with an increased number of fetuses (*p* = 0.028). However, no associations were observed for oocyte number, embryo quality, clinical pregnancy, or live birth rates.
7. Finally, Noli et al. (2023) [46] found that lower scores in adherence to the Mediterranean Diet were associated with an increased risk of unexpected poor ovarian response (aOR 0.29, 95% CI 0.11–0.76).

The seven studies included in this review [40,41,42,43,44,45,46] presented mixed findings regarding the association between Mediterranean Diet adherence and ART outcomes. A synthesis of these results reveals several key patterns:

- Clinical Pregnancy and Live Birth Rates: Three studies [40,42,43] reported positive associations between higher Mediterranean Diet adherence and improved clinical pregnancy or live birth rates. The effect sizes varied, with odds ratios ranging from 1.4 (95% CI: 1.0–1.9) [40] to 2.7 (95% CI: 1.3–5.7) [42] for clinical pregnancy, and relative risks of 2.5 (95% CI: 1.3–4.8) for live births [42]. However, two studies [44,45] found no significant associations with these outcomes.
- Oocyte and Embryo Quality: The impact on oocyte and embryo quality was less clear. While Sun et al. [45] reported an increase in the number of available embryos associated with higher Mediterranean Diet scores (8.4 ± 5.26 vs. 7.4 ± 4.71, *p* = 0.028), four studies [40,42,44,45] found no significant effects on oocyte and embryo number or quality.
- Ovarian Response: Noli et al. [46] uniquely focused on ovarian response, finding that lower adherence to the Mediterranean Diet was associated with an increased risk of poor ovarian response (aOR 0.29, 95% CI: 0.11–0.76).
- Age-Specific Effects: Some studies noted age-specific effects. For instance, Karayiannis et al. [42] found significant associations only in women under 35 years of age, while Ricci et al. [44] noted a marginally lower risk of failure to achieve clinical pregnancy for intermediate Mediterranean Diet scores in women over 35 years.
- Dose–Response Relationship: Gaskins et al. [43] observed a non-linear relationship, with improvements in live birth rates above the first quartile of adherence, but no further improvement above the second quartile.

**Table 2:** The Clinical Correlation of the Mediterranean Diet with Assisted Reproduction.

Study	Number/Quality of Oocytes	Number/Quality of Embryos	Clinical Pregnancy	Live Births
Vujkovic et al. (2010) [40]	No correlation	No correlation	OR 1.4, 95% CI 1.0–1.9	Not evaluated
Twigt et al. (2012) [41]	Not evaluated	Not evaluated	65% increase in ongoing pregnancy with 1 unit increase in nutrition score	Not evaluated
Karayiannis et al. (2018) [42]	No correlation	No correlation	RR 1.98, 95% CI 1.05–3.78	RR 2.64, 95% CI 1.37–5.07
Gaskins et al. (2019) [43]	Not evaluated	Not evaluated	Improvement in live births above the first quartile following the Mediterranean Diet	Not evaluated
Ricci et al. (2019) [44]	No correlation	No correlation	No correlation	No correlation
Sun et al. (2019) [45]	No correlation	Increased number with higher nutrition score (*p* = 0.028)	No correlation	Not evaluated
Noli et al. (2023) [46]	Increased poor response with lower nutrition score	Not evaluated	Not evaluated	Not evaluated

In all studies, the results were statistically processed for potential confounders, which are referred for each study in Table 1.

## 4. Discussion

This literature review aimed to investigate the role of compliance with the Mediterranean Diet in assisted reproduction. More specifically, the contribution of adherence to the Mediterranean Diet to the period before treatment with ART cycles, to the intermediate components of assisted reproduction techniques, as well as to the final outcome, i.e., pregnancy (biochemical or clinical) and live births [8,9]. Given the heterogeneity of the included studies in terms of study design, Mediterranean Diet assessment methods, and ART protocols, a quantitative meta-analysis was not feasible. Instead, a narrative synthesis of the findings was conducted, summarizing the key results and identifying patterns across studies. This narrative review is distinguishable for its holistic approach to the Mediterranean Diet, examining it as an indivisible dietary pattern rather than focusing on its individual components. Unlike many studies that isolate specific foods or nutrients, this review considers the synergistic interactions of the various elements within the Mediterranean Diet and their collective impact on assisted reproduction outcomes. This comprehensive perspective allows for a more nuanced understanding of how the Mediterranean Diet, as a complete dietary pattern, may influence fertility and ART success rates.

Thus, the seven included studies [40,41,42,43,44,45,46], which are likely the only original research studies that have investigated the association of pre-treatment adherence to the Mediterranean Diet to treatment with ART cycles, led to ambiguous conclusions with an overall positive sign. In total, the findings of the studies indicated a modest improvement in clinical pregnancy rates and live births [40,42,43] when there was a higher score in adherence to the Mediterranean Diet, but this association was neither linear nor proportional [43,46]. This practically indicates the existence of a threshold (cut-off point) of compliance—a score above which no additional benefits are observed in the results of assisted reproduction with a more faithful adherence to the Mediterranean Diet. In contrast, in the interim results of ART, only the study by Sun et al. (2019) [45] found that higher Mediterranean Diet scores were associated with an increased number of fetuses (*p* = 0.028). Noli et al. [46] pointed out in their cross-sectional study that reduced adherence to the Mediterranean Diet led to a poor response to ovarian stimulation and a reduced number of eggs per ovulation (≤3 mature eggs per ovulation). These considerations lead to the conclusion that the effective action of the Mediterranean Diet lies in optimizing the receptivity of the endometrium [40], implantation, smooth placentation, and later in the support and development of pregnancy due to its high nutritional value [50,51,52]. Possible components in the mechanism of these beneficial effects are the anti-inflammatory [36] and antioxidant properties [32,33,34,35,36] of plant foods, plant fibers, and unsaturated fatty acids ω3 and ω6 of the Mediterranean Diet [1,2,3,13,32,33,34,35,36,53,54,55] which favor the development of an appropriate endometrial microenvironment.

However, the positive effect of the Mediterranean Diet on the final outcome of assisted reproduction was not shown by all the studies, and in fact, some found the absence of a statistically significant correlation [44,45]. These differences can be attributed to the heterogeneity in the size of the studies, to the individual characteristics of the examined infertile women, to the ability to limit errors from confounders, and in particular, to the great heterogeneity in the methods of evaluating the adherence to the Mediterranean Diet, which as their final calibration was indicatively based on 6 to 195 different food items and groups. The various Mediterranean Diet scoring systems used across studies show some differences in their assessment methods. For instance, the MedDietScore [47] and the Mediterranean Diet Score (MDS) [1] are two commonly used tools, but they are not identical. Decarli et al. [49] conducted a comparative analysis of these two scoring systems and found an agreement rate of approximately 65%. This means that while there is substantial overlap in how these tools assess Mediterranean Diet adherence, there are also notable differences. The MedDietScore, developed by Panagiotakos et al. [47], uses an 11-component, 55-point scale that assesses the frequency of consumption of key food groups. On the other hand, the MDS, introduced by Trichopoulou et al. [1], uses a 9-component, 9-point scale based on median intakes as cut-off points. These differences in scoring methods can lead to variations in how dietary patterns are classified, which may partly explain some of the inconsistencies observed across studies. The 65% agreement rate suggests that while these tools are broadly measuring the same dietary pattern, there is room for discrepancies that could affect study outcomes. This underscores the need for standardization in Mediterranean Diet assessment tools in future research, particularly in the context of fertility and ART studies.

The positive associations observed between Mediterranean Diet adherence and ART outcomes may be explained by several potential mechanisms. The diet’s rich antioxidant content from fruits, vegetables, and olive oil could reduce oxidative stress, potentially improving gamete quality and embryo development [23,24,25,26,27,28,32,33,34,35,36]. Its anti-inflammatory properties might create a more favorable environment for implantation and early pregnancy development [36]. The Mediterranean Diet’s positive effects on glucose metabolism could influence reproductive function [13], while its emphasis on unsaturated fats, particularly omega-3 fatty acids, may support optimal oocyte and embryo development [29,30,31].

However, the absence of significant associations in some studies [44,45] could be attributed to methodological differences in diet assessment, population variations in baseline dietary habits and genetic background, timing of dietary assessment, and potentially underpowered sample sizes. These factors highlight the complexity of studying dietary impacts on fertility and the need for standardized approaches.

The potential influence of confounding factors on the reported associations is a crucial consideration. Age emerges as a potential effect modifier, with several studies finding age-specific effects [42,44]. Other important confounders include BMI, smoking and alcohol consumption, physical activity, underlying infertility diagnosis, and variations in ART protocols [40,41,42,43,44,45,46]. While most studies adjusted for these factors, residual confounding cannot be ruled out. The complex interplay between these factors and diet presents a significant challenge in interpreting the results and underscores the need for carefully designed studies that can disentangle these relationships. 

### 4.1. Potential Applications

Although the results so far are conflicting and do not demonstrate a clear and catalytic effect of the Mediterranean Diet on assisted reproductive techniques [8,9] and their outcomes, it is a fact that the Mediterranean Diet is the most popular beneficial dietary pattern for human health and fertility promotion [5,13,37,54,55]. It is therefore considered appropriate to properly inform infertile couples who resort to assisted reproduction methods about the possible beneficial actions of the Mediterranean Diet and to encourage them to comply with it, but also to participate in relevant research programs with the aim of improving the existing knowledge arsenal against infertility and the progress of IVF science. Clinicians of assisted reproduction can offer pre-treatment nutritional counseling to prospective infertile couples and recommend the Mediterranean Diet as the preferable one, after the patients’ dietary habits have been previously examined and evaluated with valid and certified questionnaires (FFQs). Optimizing candidates’ adherence to the Mediterranean Diet and its outcomes in ART cycles can be supported by the provision of individualized dietary plans (adapted to the Mediterranean Diet standards) by appropriate nutritionists, based on personal preferences and cultural background, as well as possible appropriate dietary restrictions (diabetic patients, for instance). Decision-making on the part of patient candidates needs to be based on full information about the possible benefits of the Mediterranean Diet on their reproductive potential, but also about the limitations that are set, so that the goals remain at realistic levels.

### 4.2. Limitations and Areas for Future Research

It is particularly important to mention the limitations of the present literature review so that its findings can be interpreted in an appropriate context and at the same time to highlight possible gaps in the scientific knowledge and the opportunities for further research. In particular, out of the total of seven studies, six were cohort studies, while one was a cross-sectional study (Noli et al.) [46], which indicates from their nature the impossibility of drawing safe conclusions about the real causal relationship of the Mediterranean Diet and its possible extensions to intermediate and final results of assisted reproduction techniques. All existing studies examine the Mediterranean Diet before ART treatment and not during pregnancy, although its value during pregnancy has been previously demonstrated by multiple studies. Furthermore, the studies also showed heterogeneity in the ART protocol followed, based on the experience of each IVF center and its location. The review did not focus on the effect of the Mediterranean Diet on the sperm parameters of infertile men, which is now well known to have multiple benefits [13,14,15,16,17,18,19,20,21,22,37,56,57,58,59,60]. This poses a further important limitation as male infertility is considered to contribute equally to the infertility of reproductive-age couples [61], a fact that must be taken into consideration in the interpretation of the results. This literature review focuses only on infertile couples seeking childbearing through ART, and therefore, any beneficial effects of the Mediterranean Diet shown by the studies mentioned should not be generalized to other couples and women of reproductive age seeking pregnancy through natural conception, although other studies support their existence [18,19,20,23,24,25,26,27,28,62,63,64,65,66,67]. An important limitation is posed by the fact that the FFQs were based on the personal recall from memory of the examined women, which involves a high risk of recall bias.

In this light, future research in the field of reproductive medicine, particularly concerning the role of the Mediterranean Diet in assisted reproduction techniques, should prioritize several key areas. Firstly, well-designed randomized clinical trials (RCTs) are essential to establish causal relationships between Mediterranean Diet adherence and ART outcomes. These RCTs should ideally be multicenter, involving a larger number of participating couples, including both women and men with infertility, to increase generalizability and statistical power. Following the model of the PREPARE study [68], a follow-up period of at least 6 months is recommended to balance the need for longitudinal data with practical considerations of participant retention and compliance. Secondly, the development of objective and standardized methods for assessing Mediterranean Diet adherence is crucial. This could involve creating a validated, fertility-specific Mediterranean Diet score and implementing regular dietary assessments through a combination of digital tools, biomarker analysis, and expert consultations. A hybrid monitoring approach combining remote check-ins (e.g., via phone or digital platforms) with face-to-face meetings with nutritionists could enhance data accuracy and participant adherence. Thirdly, future studies should explore the impact of the Mediterranean Diet on various stages of the ART process, from gamete quality to implantation and early pregnancy development. This could involve mechanistic studies examining the effects of specific dietary components on reproductive biomarkers and outcomes. Moreover, research should investigate potential gene–diet interactions that may modulate the effects of the Mediterranean Diet on fertility and ART outcomes. This could lead to more personalized dietary recommendations based on individual genetic profiles. Additionally, studies should consider the long-term impacts of Mediterranean Diet adherence during ART on offspring health, extending follow-up beyond live birth rates to examine developmental outcomes in children conceived through ART. Finally, qualitative research exploring barriers and facilitators to Mediterranean Diet adherence in couples undergoing ART could inform the development of targeted interventions to support dietary changes in this population.

By addressing these research priorities, we can significantly advance our understanding of the role of the Mediterranean Diet in ART and potentially improve outcomes for couples struggling with infertility. This multifaceted approach will help mitigate confounding factors, reduce recall bias [69], and provide more robust evidence to inform clinical practices and dietary guidelines in the context of assisted reproduction.

## 5. Conclusions

In conclusion, it appears that the Mediterranean Diet is an excellent dietary pattern that promotes health and fertility, and possibly, higher adherence to it may result in improved parameters of assisted reproduction, especially clinical pregnancy and live and viable child births. However, further rigorous research by organizing randomized clinical trials is deemed imperative to concretize and weigh its contribution to assisted reproduction and to formulate appropriate public health policies and guidelines for concerned couples whose goal of creating a family with children is challenged by the infertility they face.

## Figures and Tables

**Table 1:** The included studies of the review and their key characteristics.

First Author (Year)	Country	Study Design	Sample Size and Characteristics	ART Protocol	Method of Evaluation of the Mediterranean Diet	Duration of the Study and Follow-up Period	Results	Mediterranean Diet and ART Association	Confounders
Vujkovic et al. (2010) [40]	Netherlands	Prospective Cohort	IVF/ICSI treatment at a university IVF clinic, median age of women ≈35 years, median BMI ≈23 kg/m^2^	Not clarified	- 195 questions about foods in a FFQ analyzed in terms of main components to identify dietary patterns - The evaluation of following the Mediterranean Dietary Pattern was determined with a score of 0–8	- Conducted from September 2004 to January 2007—Preconception diet of the previous 4 weeks - Follow-up after ART not clarified	- Biochemical pregnancy (+urinary β-hCG 15 days after ovulation) - Quality of embryos on the 3rd day	- High adherence to the Mediterranean Diet led to an increase in the probability of pregnancy by 1.4 times although not statistically significant (OR 1.4, 95% CI 1.0–1.9) - No correlation found between embryo quality and Mediterranean Diet	- BMI, Smoking, Alcohol, IVF/ICSI Therapy, Ovarian Stimulation Protocol
Twigt et al. (2012) [41]	Netherlands	Prospective Cohort	199 women undergoing 1st IVF/ICSI cycle at a university IVF clinic	Not clarified	- 6 questions about the frequency of intake of fruits, vegetables, meat, fish, whole grain products, and fatty foods. - Preconception nutritional risk score [preconception dietary risk (PDR) score] with higher score = better diet quality	- Conducted from October 2007 to October 2010—Pre-Conception Diet - Follow-up after ART not clarified	- Pregnancy development (Ultrasound detection of fetal heart rate at the 10th week of gestation)	- 1 unit increase in PDR score increased the odds of pregnancy progression by 1.65 (aOR 1.65, 95% CI 1.08–2.52)	- Woman’s Age, Smoking, Partner’s PDR Score, Couple’s BMI, ART Treatment Indication
Karayiannis et al. (2018) [42]	Greece	Prospective Cohort	244 non-obese women aged 22–41 years with BMI < 30 kg/m^2^ undergoing 1 IVF cycle (ICSI) in a private IVF clinic. Mainly infertile couples of male etiology	GnR H agonist protocol—rFSH and/or hMG at a max combined dose of 450 IU/day	- 76-point Mediterranean Diet score from 0–55 that assessed intake of foods from 10 food groups	- Held from 2013 to 2016 - Pre-conception diet - Follow-up after ART not clarified	- Oocytes retrieved, mature oocytes, fertilisation rate, embryo quality at day 3, clinical pregnancy, live births [8,9]	- Clinical Pregnancy: 50% in the upper tertile (MedDietScore ≥36, *n* = 86) vs. 29% in the lower tertile (MedDietScore ≤30, *n* = 79), *p* = 0.01 - Live births: 49% in upper tertile vs. 27% in lower tertile, *p* = 0.01 - Differences in clinical pregnancy and live births were found only in women <35 years of age - No significant differences were found in the other results	- Age, Ovarian Stimulation Protocol, BMI, Physical Activity, Stress, Infertility Diagnosis, Caloric Intake, Supplement Use
Gaskins et al. (2019) [43]	USA	Prospective Cohort	357 women aged 31–39 years and BMI: 21–28 who underwent a total of 608 cycles of ART treatment	Multiple Protocols	- Mediterranean Diet Score from 0–55 which assessed intake of foods from 11 food groups	- Evaluation of data from 2007 to 2017 - Pre-conception diet - Women were followed for 1 (55%), 2 (26%), 3 (13%) 9, or 4–6 cycles of ART (5%)	- Live births - Clinical Pregnancy	- The Mediterranean Diet was found to improve live birth rates above the first quartile of adherence (0.44, 95% CI: 0.39–0.49, *p* < 0.05) while in the first quartile: (0.31, 95% CI: 0.25–0.39, *p* < 0.05) - However, there was no further improvement in live birth rates above the second quartile - No significant correlation was noted with clinical pregnancy rates	- Age, BMI, Caloric Intake, Physical Activity, Smoking, Infertility Diagnosis, Previous Pregnancies, Training, Dietary Supplements
Ricci et al. (2019) [44]	Italy	Prospective Cohort	474 women aged 23–40 years, with BMI: 18.3–26.3, treated with an IVF cycle in an Italian IVF clinic	Not clarified	- Mediterranean Diet Score from 0–9 that assessed intake of 9 food groups, via 78-questions of food frequency in a certified FFQ	- Held from September 2014 to December 2016 - Pre-conception diet - Follow-up after ART not clarified	- Retrieved Eggs, Quality and Number of Embryos on Day 2/3, Embryo Transfer, Clinical Pregnancy, Live Births	- No significant association of the Mediterranean Diet Score with oocyte count, embryo quality, clinical pregnancy or live births emerged - Minimally lower risk of failure to achieve clinical pregnancy for intermediate Mediterranean Diet Score in women >35 years (aRR 0.84, 95% CI 0.71–1.00, *p* < 0.05) with no associated increase in live birth rates	- Age, Physical Activity, BMI, Smoking, Daily Caloric Intake, Previous ART Cycles
Sun et al. (2019) [45]	China	Prospective Cohort	590 infertile women aged 28–35.5 years undergoing IVF treatment	- Long GnRH agonist or antagonist protocol	- Mediterranean Diet Score from 0–8 that assessed the intake of 8 food groups (alcohol removed), via a 69-question food frequency non-validated FFQ	- Held from September 2016 to December 2017 - Pre-conception diet - Follow-up only for Embryo Transfer	- Retrieved Eggs, Number of Embryos, Quality of Embryos on Day 3, Clinical Pregnancy, Implantation	- Higher Mediterranean Diet Score led to an increase in the number of available embryos (8.4 ± 5.26 vs. 7.4 ± 4.71, *p* = 0.028) - No significant differences emerged in the other results under examination	- Age, Infertility Diagnosis, BMI, Basal FSH, Duration and dose of Gonadotropins
Noli et al. (2023) [46]	Italy	Cross-Sectional	296 infertile women aged 19–39 years, with normal BMI and ovarian reserve, undergoing IVF treatment	- Long-acting GnRH agonist or antagonist protocol - Initial dose of gonadotropins 150–225 IU/day	- Mediterranean Diet Score from 0–9 that assessed intake of 9 food groups, via 78-questions of food frequency in a certified FFQ	- Held from September 2014 to February 2019 - Pre-conception diet - No follow-up was performed after ART, as a cross-sectional study that is a snapshot of data in a specific time period	- Unexpected poor ovarian response after stimulation (≤3 mature eggs in ovulation)	- Low Mediterranean Diet Score led to an increased risk of Unexpected poor response, statistically significant association especially for the middle tertile of the Mediterranean Diet Score versus the lower tertile: aOR 0.29 (95% CI 0.11–0.76) - For middle and upper tertile women combined vs. lower: aOR 0.34 (95% CI 0.14–0.82)	- Age, BMI, Smoking, Endometriosis, Caloric Intake, Alcohol, Caffeine

ART: Assisted reproduction techniques, ICSI: Intra-cytoplasmic sperm inclusion, IVF: In vitro Fertilization, FFQ: Food Frequency Questionnaire, OR: Odds Ratio, aOR: advance Odds Ratio, aRR: Risk Ratio, CI: Confidence Interval.
